# Designing Emotions for Health Care Chatbots: Text-Based or Icon-Based Approach

**DOI:** 10.2196/39573

**Published:** 2022-12-08

**Authors:** Shubin Yu, Luming Zhao

**Affiliations:** 1 Department of Communication and Culture BI Norwegian Business School Oslo Norway; 2 School of Journalism Fudan University Shanghai China

**Keywords:** chatbot, health care, emotion, psychological distance, perception, human behavior, behavioral intention, predict, emotional intensity, text-based, icon-based, design

## Introduction

Health care chatbots, which are being widely adopted by providers, offer many benefits to users [1]. However, the limited communication capabilities of chatbots hinder their interactions with humans [2]. Therefore, text-based (ie, verbal emotional expression, eg, saying “I am so sorry to hear that”) and icon-based (ie, nonverbal emotional expression, eg, using emojis, emoticons, or stickers) approaches are adopted to communicate emotion in chatbot messages. Previous studies have suggested that both emotion design approaches are effective in improving the evaluation of health care chatbots [3,4]. However, the two approaches differ greatly from each other in their presentation, mechanism, and effectiveness. Understanding such differences could help system developers to optimize their health care chatbots. Nevertheless, research comparing these two approaches of emotion designs, to our knowledge, is nonexistent. This study aims to understand the mechanism and the interaction effect of these two approaches to see if the effect of one approach depends on the other one. In general, we proposed the following hypothesis: both text-based and icon-based emotional clues for health care chatbots can increase perceived emotional intensity (H1). To test the interaction effect of the two approaches, we hypothesized that the addition of an icon-based clue would not significantly affect emotional intensity when a text-based clue is already present (H2). Furthermore, emotional intensity will reduce psychological distance and increase behavioral intention (H3). Please refer to Multimedia Appendix 1 for the theoretical framework and hypothesis development.

## Methods

In total, 483 respondents were recruited through a web-based panel in China. The mean age of the participants was 28.8 (SD 8.84) years. A majority of participants self-identified as female (n=300, 62.1%). We used a 2 (text-based emotion design: yes vs no) by 2 (icon-based emotion design: yes vs no) between-subjects factorial experimental design. Participants were asked to imagine they had abdominal pain and then consult a prediagnostic chatbot online. The participants were randomly assigned to one of the four conditions. They were shown a screenshot of a conversation with the chatbot. After viewing the screenshot, participants were asked to answer a series of questions about perceived emotional intensity, psychological distance, and behavioral intention (see Multimedia Appendix 1).

## Results

The results of a 2-way ANOVA showed that both icon-based (mean_icon_ 4.15, SD 1.41; mean_no_ 3.62, SD 1.44; *F*_1,479_=14.4; *P*<.001; *η*^2^=0.03) and text-based designs significantly enhanced the perceived emotional intensity (mean_text_ 4.35, SD 1.38; mean_no_ 3.46, SD 1.40; *F*_1,479_=51.2; *P*<.001; *η*^2^=0.10). H1 was therefore supported. Furthermore, we observed an interaction effect between icon- and text-based designs (*F*_1,479_=7.96; *P*=.006; *η*^2^=0.02). In particular, when text-based designs were not used, icon-based designs increased the emotional intensity (mean_icon_ 3.87, SD 1.39; mean_no_ 3.05, SD 1.26; *F*_1,243_=23.2; *P*<.001; *η*^2^=0.09). However, when text-based designs were used, the effect of icon-based designs disappeared (mean_icon_ 4.41, SD 1.40; mean_no_ 4.29, SD 1.35; *F*_1,236_=0.45; *P*=.50; *η*^2^=0.002). These findings were consistent with H2.

**Figure 1:**
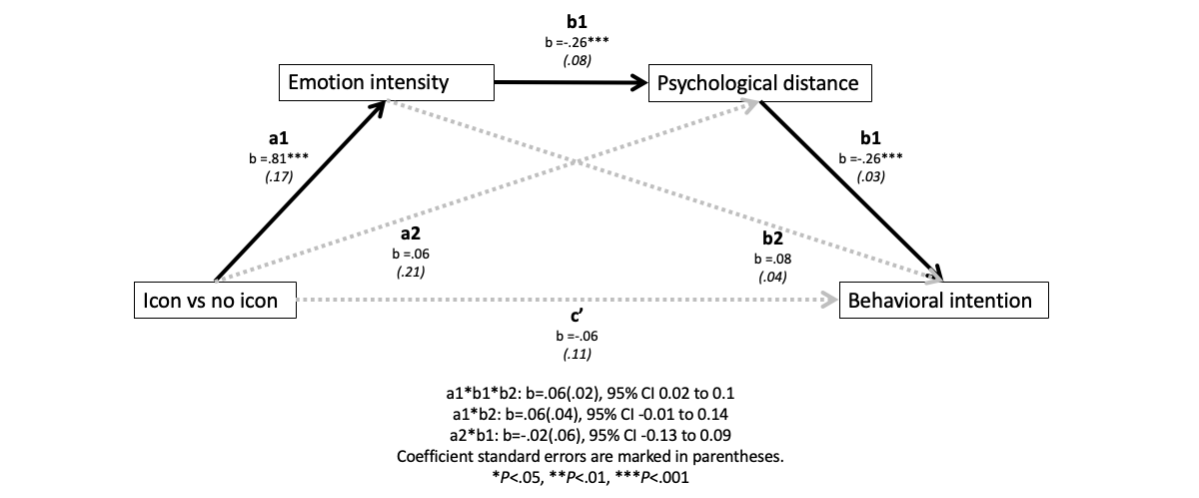
The indirect effect of icon-based designs when text-based designs were not used.

**Figure 2:**
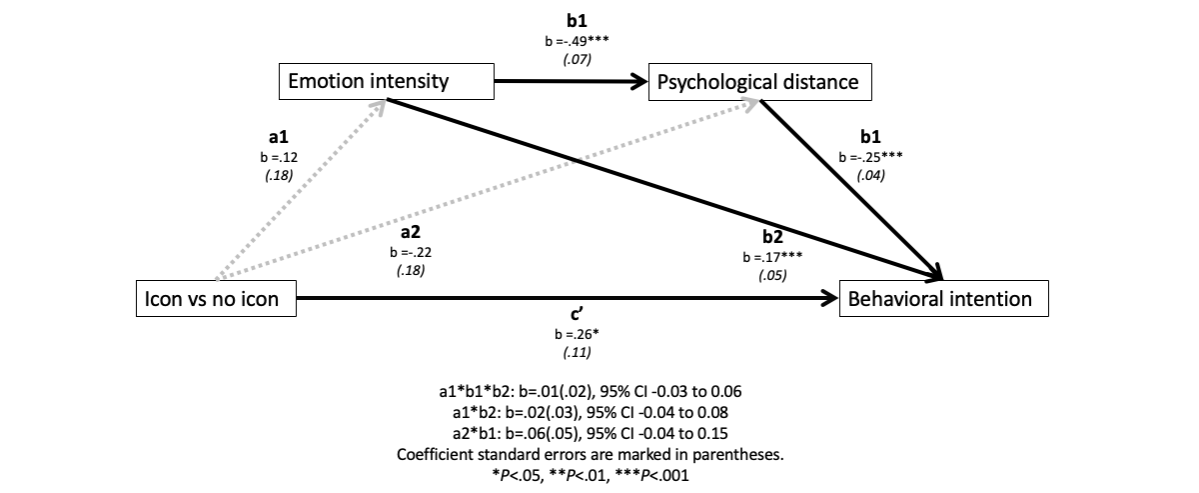
The indirect effect of icon-based designs when text-based designs were used.

We performed a moderated serial mediation model to further test our hypotheses. The analysis revealed a significant moderated mediation index (effect −0.07, SE 0.03, lower limit CI −0.12, upper limit CI −0.02). When there was no text-based design, icon-based designs significantly increased the emotional intensity, and thus shortened the psychological distance and enhanced the behavioral intention (Figure 1). This indirect effect was not significant when text-based designs were used (Figure 2). Overall, H3 was supported.

## Discussion

This study demonstrated that both icon- and text-based emotion designs of health care chatbots increase users’ perceived emotional intensity. The effect of the text-based approach on emotional intensity was stronger than the icon-based one. The impact of the icon-based design on the perceived emotional intensity was mitigated when combining text-based designs together. Furthermore, the perceived emotional intensity reduced the psychological distance and enhanced behavioral intention. This is consistent with previous studies on interpersonal emotion disclosure and psychological distance [5].

The findings fill a void in the literature on health care chatbots’ emotional design. In particular, we observed an antagonist effect of the two approaches of emotion design on emotional intensity, suggesting that using a single approach is sufficient. Additionally, previous research has examined the psychological distance between physical social robots and humans [6,7]. This study extends our understanding of the role of psychological distance in the effect of emotional expression of health care chatbots. Future studies may also investigate when the use of icon-based designs may backfire (eg, when conveying different emotional valence [8]). For example, the use of emojis may make interactions appear unprofessional in contexts like medical consultation [9]. This implies that the use of icon-based emotional designs may have negative effects under certain circumstances for health care chatbots, which should be further explored in future research (see Multimedia Appendix 1 for details).

## Appendix

Multimedia Appendix 1 Supplementary information.
