# Supplementary material for: Designing Emotions for Health Care Chatbots: Text-Based or Icon-Based Approach
Source: J Med Internet Res. 2022 Dec 8;24(12):e39573. doi: 10.2196/39573 (PMC9782388; doi:10.2196/39573)
Supplement: Multimedia Appendix 1 [file jmir_v24i12e39573_app1.docx]

**SUPPLEMENTARY MATERIALS**

1. **Table 1. Demographic information of the participant**

| Variables | Classification | Number | Percentage |
| --- | --- | --- | --- |
| Gender | Male | 183 | 37.9 |
|  | Female | 300 | 62.1 |
| Occupation | Students | 199 | 41.2 |
|  | Civil servants | 23 | 4.8 |
|  | Employees | 181 | 37.5 |
|  | Workers | 14 | 2.9 |
|  | Service providers | 7 | 1.4 |
|  | Specialists | 32 | 6.6 |
|  | Freelancers | 16 | 3.3 |
|  | Others | 11 | 2.3 |
| Educational background | Less than high school | 6 | 1.2 |
|  | High school | 14 | 2.9 |
|  | Bachelor (College) | 37 | 7.7 |
|  | Bachelor (University) | 280 | 58.0 |
|  | Master’s degree | 146 | 30.2 |

1. **The script and screenshot**^[[1]](#footnote-1)^

To develop the experimental material, we have studied extensively virtual agent services deployed by Chinese technology companies such as Ping An, Baidu, and Alibaba on healthcare platforms. For Chinese healthcare chatbots, companies utilize names like “智能小护士” or “智能医疗助手” (both mean AI healthcare assistant). In this study, we used the name “智能预分诊小护士” (AI pre-diagnosis healthcare assistant) for the healthcare chatbot.

Here is the scenario for the experiment: Imagine that you experienced abdominal pain recently. The abdominal pain lasted for several days. You were too busy to go to the hospital. Therefore, you decided to consult an AI-powered pre-diagnosis chatbot.

| Without both text-based and icon-based emotional designs | With text-based and without icon-based emotional designs |
| --- | --- |
| Hello, I am an AI pre-diagnosis healthcare assistant. What can I do for you? | Hello, I am an AI pre-diagnosis healthcare assistant. I am very happy to help you. |
| I have abdominal pain. | I have abdominal pain. |
| I have registered your problem. Which kind of symptoms did you experience? | Oh no, I am so sorry to hear that. Which kind of symptoms did you experience? |
| Continuous cramp-like pain | Continuous cramp-like pain |
| It is likely to be due to gas and bloating. Did you experience diarrhoea or fever? | It is likely to be due to gas and bloating. Did you experience diarrhoea or fever? |
| No | No |
| I see. What other symptoms do you have? | I see. What other symptoms do you have? |
| A little bit of nausea. | A little bit of nausea. |
| How long have the symptoms lasted? | How long have the symptoms lasted? |
| One week | One week |
| Your disease is probably gastritis. You could go to the Department of Gastroenterology for a formal diagnosis and further treatment.  Meanwhile, you could drink more warm water and take sufficient rest. Please also avoid alcohol. | Your disease is probably gastritis. Don’t worry too much. You could go to the Department of Gastroenterology for a formal diagnosis and further treatment.  Meanwhile, you could drink more warm water and take sufficient rest. You should try to avoid alcohol, OK? |
| Thanks | Thanks |
| 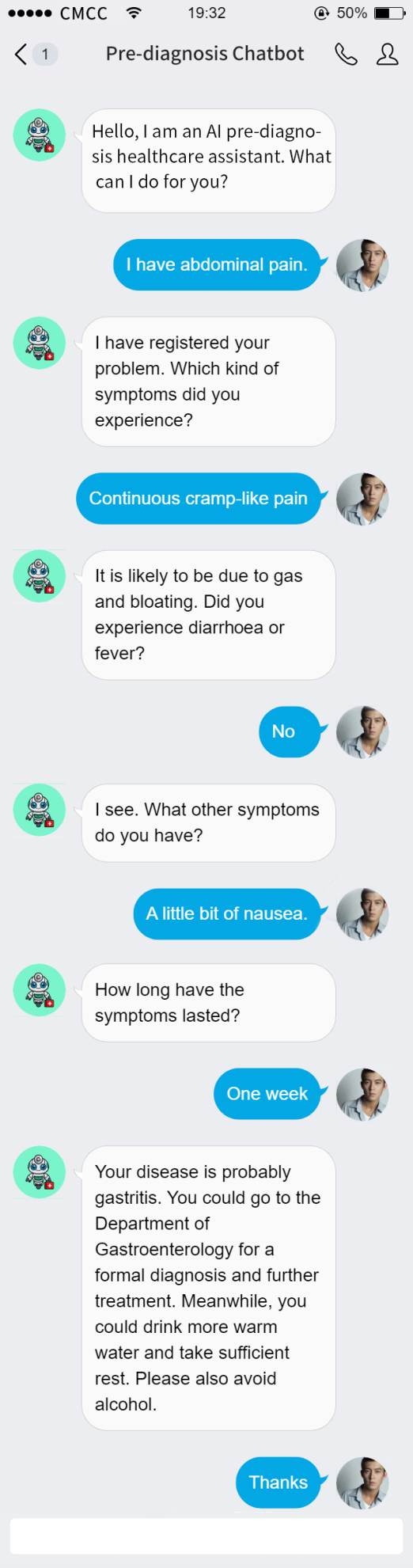 | 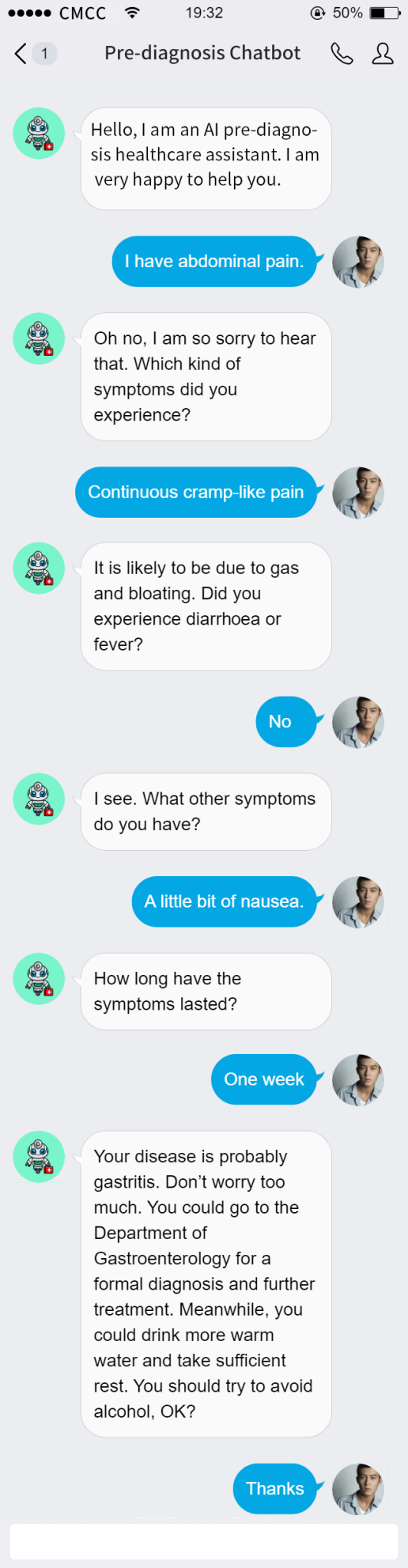 |

| Without text-based and with icon-based emotional design | With both text-based and icon-based emotional design |
| --- | --- |
| Hello, I am an AI pre-diagnosis healthcare assistant. What can I do for you?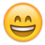 | Hello, I am an AI pre-diagnosis healthcare assistant. I am very happy to help you.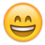 |
| I have abdominal pain. | I have abdominal pain. |
| 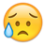 I have registered your problem. Which kind of symptoms did you experience? | 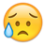 Oh no, I am so sorry to hear that. Which kind of symptoms did you experience? |
| Continuous cramp-like pain | Continuous cramp-like pain |
| It is likely to be due to gas and bloating. 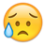Did you experience diarrhoea or fever? | It is likely to be due to gas and bloating. 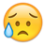 Did you experience diarrhoea or fever? |
| No | No |
| I see. What other symptoms do you have? | I see. What other symptoms do you have? |
| A little bit of nausea. | A little bit of nausea. |
| How long have the symptoms lasted? | How long have the symptoms lasted? |
| One week | One week |
| Your disease is probably gastritis. You could go to the Department of Gastroenterology for a formal diagnosis and further treatment.  Meanwhile, you could drink more warm water and take sufficient rest. Please also avoid alcohol. 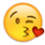 | Your disease is probably gastritis. Don’t worry too much. You could go to the Department of Gastroenterology for a formal diagnosis and further treatment.  Meanwhile, you could drink more warm water and take sufficient rest. You should try to avoid alcohol, OK? 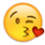 |
| Thanks | Thanks |
| 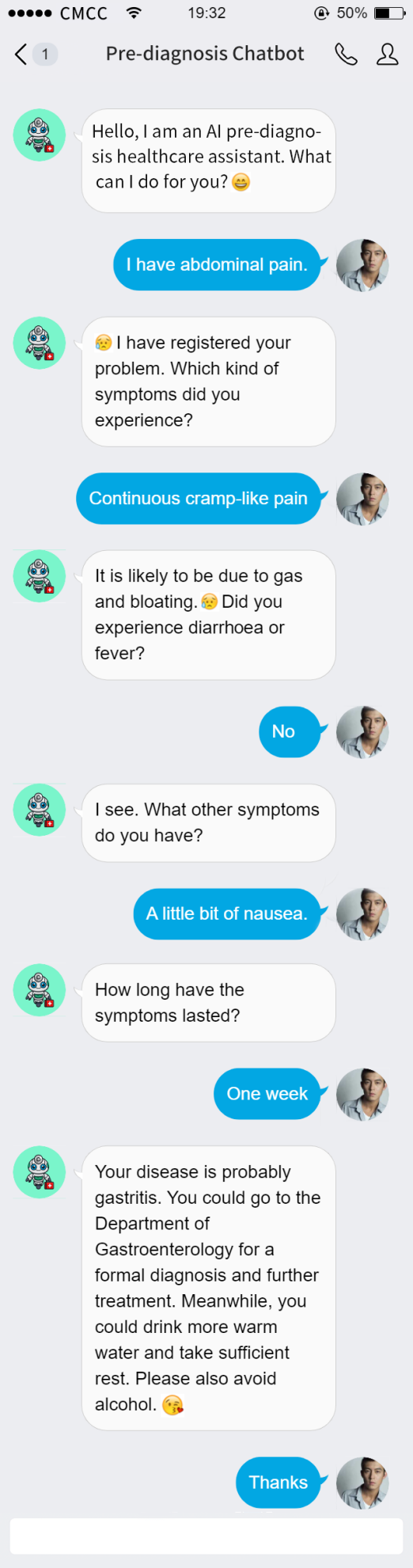 | 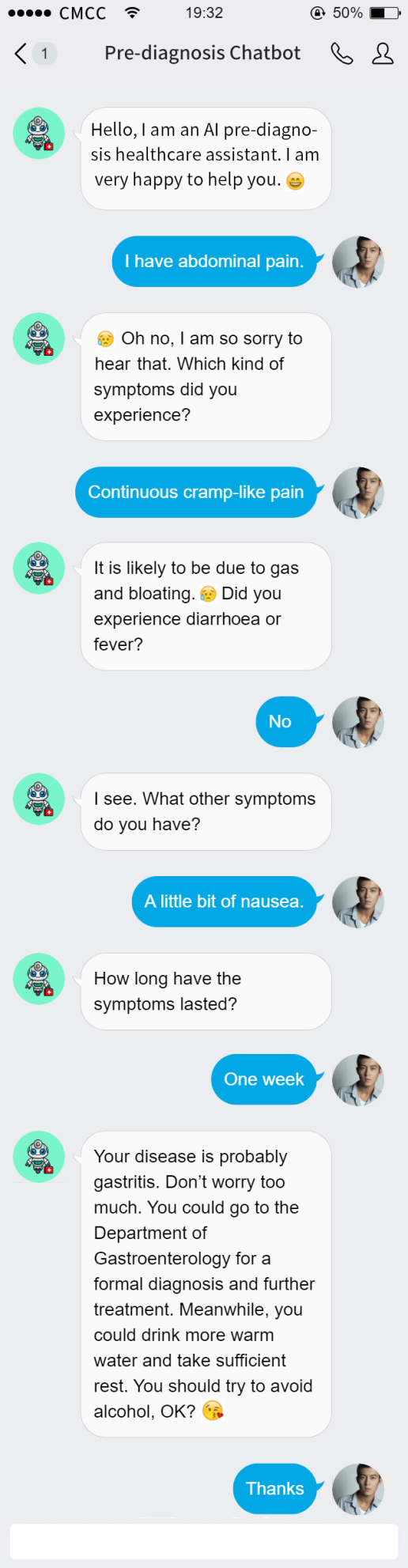 |

1. **Theoretical framework and hypothesis development**

**CASA and perceived emotion intensity**

Human interactions with chatbots produce emotional responses similar to interpersonal interactions, based on the paradigm that computers are social actors (CASA; Reeves & Nass, 1996). CASA suggests that people treat media, such as computers, with the same judgments and reactions as they do other humans (Eyssel & Frank, 2012; Lombard & Xu, 2021), and this tendency is thought to occur unconsciously (Langer, 1992). Recent studies have confirmed the effectiveness of CASA in explaining computerized agents in different dimensions (Annabell et al., 2018). For example, Ho et al. (2018) noted that participants' self-representations of their chatbot partners produced similar emotional, relational, and psychological benefits to those of their human partners. This implies that emotional expressions of chatbots can influence users’ perceptions and responses. However, the way chatbots express emotions is usually text-based or icon-based. The in-text language style can be considered an important design feature that influences the perception of chatbots. By using different language styles, chatbots can give instructions and information in an authoritative or a sympathetic manner (Gnewuch, 2020; Al-Natour, 2006). Apart from the verbal approach, developers could also show iconic cues to manipulate the emotion of chatbots, such as emojis or emoticons. Prior CMC literature suggests that visual cues like emojis assist the human–computer interaction, including clarifying intentions in high contexts, reducing uncertainty, and modulating the affective impact of messages (Rodrigues et al., 2017; Aldunate & Gonzalez-Ibanez, 2017; Huang et al., 2021; Derks et al., 2008a, 2008b). We hypothesized (H1) that both icon-based and text-based designs would enhance the perceived emotional intensity of the healthcare chatbot. However, we expected there will be an antagonist effect of the two approaches on emotional intensity. In other words, the presence of text-based emotion design may impede the positive effect of icon-based design on emotional intensity because text-based emotion design may already elicit a rather high level of emotional intensity.

Previous studies suggest that people perceive others’ emotions not only based on visual elements like facial expressions but also on verbal elements like words (Lindquist & Gendron, 2013). In particular, the effect of the visual element on emotion perception depends greatly on the verbal elements (Lindquist & Gendron, 2013). In the context of using a pre-diagnosis healthcare chatbot, patients generally have higher service involvement. In this case, when processing information from the chatbot, they may enter the central (vs. peripheral) processing route and thus rely more on the text than the visual cues. In this case, the effect of the icon-based emotion design on emotional intensity may be less prominent when text-based emotion design is present (H2).

**Psychological distance**

Showing emotion or emotional self-disclosure can increase perceived intimacy (Laurenceau et al., 2004) and reduce psychological distance. Psychological distance is a concept that defines how close or far an object is psychologically and is determined by time, space, probability, and social distance between the self and the object/others (Trope & Liberman 2010). Similarity is an important attribute that determines psychological distance. Previous studies have found that individuals feel psychologically close to people with similar attributes (Liviatan et al., 2008; Tajfel, 1982). As chatbots are perceived to be quite dissimilar from human agents, one could expect that the psychological distance of chatbots could be large. This explains the reason that many users remain uncomfortable or hesitant with chatbots (Zamora, 2017; Luo et al., 2019). If chatbots can express emotion, one may perceive them as more human-like. The human-likeness of chatbots further reduces psychological distance (Li and Sung, 2021; Liu & Sundar, 2018). Additionally, the level of interpretation theory suggests that the psychological distance from the target affects the way individuals interpret it. When the target is similar to them, individuals perceive the target's behavior as more specific and adopt a positive evaluation (Liviatan et al., 2008). We, therefore, hypothesized that emotional intensity would further reduce the psychological distance between the user and the chatbot, thus enhancing the user's behavioral intention (H3).

1. **Limitations**

This study has certain limitations, which point, in turn, to avenues for future research. First, our experiment was conducted in one country, China. Expressions of emotion and interpretation of emojis may be culturally bound, suggesting that findings from this study may not generalize to other culturally different areas completely. Cultural background (high vs. low context) could influence the effect of either the text- or icon-based design approach. Second, the experimental scenario is limited. Our experiments were designed for a relatively urgent disease situation, and the results may be different for some chronic disease scenarios. Third, this study has confirmed the positive effect of the use of emojis when communicating with patients. Future studies may also investigate when and how (based on the effect of different emotional valence, Kaye et al., 2021) the use of emojis may backfire. For example, the use of emojis may make the interaction casual and unprofessional, as suggested by Fadhil et al. (2018): Participants scored lower on chatbots that used emojis when asking for information about their physical well-being compared to the plain text dialogue. This implies that the inclusion of icons (emoticons) in the emotional design of healthcare chatbots is still a debated issue.

1. **Table 3. Measurement**

| **Construct** | **Item** | **Statements** | **Source** |
| --- | --- | --- | --- |
| Perceived emotion intensity | emo1 | The chatbot showed emotions. | Cheshin et al. (2018)  α = .92 |
|  | emo2 | I can perceive the emotions of the robot. |  |
|  | emo3 | The emotion expressed by the robot is strong. |  |
| Psychological distance | Please select the best picture representing the relationship between you and the chatbot. The green circle represents you and the red circle represents the chatbot.  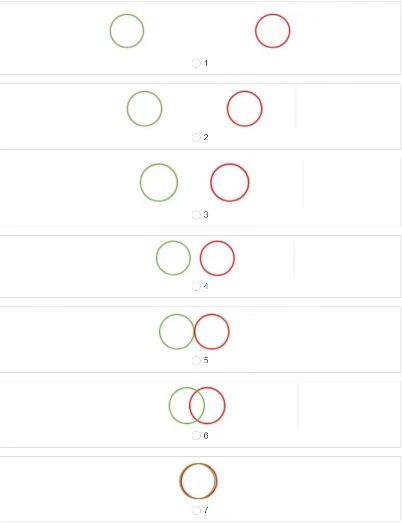 | | Berscheid et al. (1989) |
| Behavioral intention | BI1 | I will follow the advice of this healthcare chatbot. | α = .81 |
|  | BI2 | I will use this healthcare chatbot in the future. |  |
|  | BI3 | I will recommend this healthcare chatbot to other people. |  |

1. **References for supplementary materials**

Aldunate, Nerea, and Roberto González-Ibáñez. "An integrated review of emoticons in computer-mediated communication." *Frontiers in psychology* 7 (2017): 2061.

Al-Natour, Sameh, Izak Benbasat, and Ronald T. Cenfetelli. "The role of design characteristics in shaping perceptions of similarity: The case of online shopping assistants." *Journal of the Association for Information Systems* 7.12 (2006): 34.

Annabell, Lucas, et al. "Hip pathology: the diagnostic accuracy of magnetic resonance imaging." *Journal of Orthopaedic Surgery and Research* 13.1 (2018): 1-5.

Derks, Daantje, Agneta H. Fischer, and Arjan ER Bos. "The role of emotion in computer-mediated communication: A review." *Computers in human behavior* 24.3 (2008): 766-785.

Derks, Daantje, Arjan ER Bos, and Jasper Von Grumbkow. "Emoticons in computer-mediated communication: Social motives and social context." *Cyberpsychology & behavior* 11.1 (2008): 99-101.

Eyssel, Friederike, and Frank Hegel. "(s) he's got the look: Gender stereotyping of robots 1." *Journal of Applied Social Psychology* 42.9 (2012): 2213-2230.

Fadhil, Ahmed, et al. "The effect of emojis when interacting with conversational interface assisted health coaching system." *Proceedings of the 12th EAI international conference on pervasive computing technologies for healthcare*. 2018.

Gnewuch, Ulrich, Meng Yu, and Alexander Maedche. "The effect of perceived similarity in dominance on customer self-disclosure to chatbots in conversational commerce." *Proceedings of the 28th European Conference on Information Systems (ECIS 2020)*. 2020.

Ho, Annabell, Jeff Hancock, and Adam S. Miner. "Psychological, relational, and emotional effects of self-disclosure after conversations with a chatbot." *Journal of Communication* 68.4 (2018): 712-733.

Huang, Yingying, et al. "Interactivity in online chat: Conversational cues and visual cues in the service recovery process." *International Journal of Information Management* 60 (2021): 102360.

Kaye, Linda K., et al. "How emotional are emoji?: Exploring the effect of emotional valence on the processing of emoji stimuli." *Computers in Human Behavior* 116 (2021): 106648.

Langer, Ellen J. "Matters of mind: Mindfulness/mindlessness in perspective." *Consciousness and cognition* 1.3 (1992): 289-305.

Laurenceau, Jean-Philippe, et al. "Intimacy as an interpersonal process: Current status and future directions." *Handbook of closeness and intimacy* (2004): 61-78.

Li, Xinge, and Sung Yongjun. "Anthropomorphism brings us closer: The mediating role of psychological distance in User–AI assistant interactions." *Computers in Human Behavior* 118 (2021): 106680.

Lindquist, K. A., & Gendron, M. (2013). What’s in a word? Language constructs emotion perception. Emotion Review, 5(1), 66-71.

Liu, Bingjie, and S. Shyam Sundar. "Should machines express sympathy and empathy? Experiments with a health advice chatbot." *Cyberpsychology, Behavior, and Social Networking* 21.10 (2018): 625-636.

Liviatan, Ido, Yaacov Trope, and Nira Liberman. "Interpersonal similarity as a social distance dimension: Implications for perception of others’ actions." *Journal of experimental social psychology* 44.5 (2008): 1256-1269.

Lombard, Matthew, and Xu Kun. "Social responses to media technologies in the 21st century: The media are social actors paradigm." *Human-Machine Communication* 2 (2021): 29-55.

Luo, Xueming, et al. "Frontiers: Machines vs. humans: The impact of artificial intelligence chatbot disclosure on customer purchases." *Marketing Science* 38.6 (2019): 937-947.

Reeves, Byron, and Clifford Nass. "The media equation: How people treat computers, television, and new media like real people." *Cambridge, UK* 10 (1996): 236605.

Rodrigues, David, et al. "A frown emoji can be worth a thousand words: Perceptions of emoji use in text messages exchanged between romantic partners." *Telematics and Informatics* 34.8 (2017): 1532-1543.

Tajfel, Henri. "Social psychology of intergroup relations." *Annual review of psychology* 33.1 (1982): 1-39.

Trope, Yaacov, and Liberman Nira. "Construal-level theory of psychological distance." *Psychological review* 117.2 (2010): 440.

Zamora, Jennifer. "I'm sorry, dave, i'm afraid i can't do that: Chatbot perception and expectations." *Proceedings of the 5th international conference on human agent interaction*. 2017.

1. The original language is Chinese. [↑](#footnote-ref-1)
